# Intrathecal gastrodin alleviates allodynia in a rat spinal nerve ligation model through NLRP3 inflammasome inhibition

**DOI:** 10.1186/s12906-024-04519-w

**Published:** 2024-06-04

**Authors:** JunXiu Jin, Dong Ho Kang, Geon Hui Lee, Woong Mo Kim, Jeong Il Choi

**Affiliations:** 1 Department of Anesthesiology and Pain Medicine, Chonnam National University Medical School and Hospital, 42 Jebong-ro, Dong-gu, Gwangju, 61469 Korea; 2 Department of Anesthesia, First Affiliated Hospital, Zhejiang University School of Medicine, Hangzhou, People’s Republic of China; 3 BioMedical Sciences Graduate Program (BMSGP), Chonnam National University Medical School, Hwasun, 58128 Korea

**Keywords:** Gastrodin, NLRP3, Inflammasome, Spinal cord, Allodynia, Spinal nerve ligation

## Abstract

**Background:**

Gastrodin (GAS), a main bioactive component of the herbal plant, Gastrodia elata Blume, has shown to have beneficial effects on neuroinflammatory diseases such as Alzheimer’s disease in animal studies and migraine in clinical studies. Inflammasome is a multimeric protein complex having a core of pattern recognition receptor and has been implicated in the development of neuroinflammatory diseases. Gastrodin has shown to modulate the activation of nucleotide-binding oligomerization domain (NOD)-like receptor protein 3 (NLRP3) inflammasome. This study investigated the effects of GAS on the intensity of mechanical allodynia and associated changes in NLRP3 inflammasome expression at the spinal level using L5/6 spinal nerve ligation model (SNL) in rats.

**Methods:**

Intrathecal (IT) catheter implantation and SNL were used for drug administration and pain model in male Sprague-Dawley rats. The effect of gastrodin or MCC950 (NLRP3 inflammasome inhibitor) on mechanical allodynia was measured by von Frey test. Changes in NLRP3 inflammasome components and interleukin-1β (IL-1β) and cellular expression were examined in the spinal cord and dorsal root ganglion.

**Results:**

The expression of NLRP3 inflammasome components was found mostly in the neurons in the spinal cord and dorsal root ganglion. The protein and mRNA levels of NLRP3, apoptosis-associated speck-like protein containing a caspase recruitment domain (ASC), caspase-1, and IL-1β were upregulated in SNL animals compared to Sham animals. IT administration of GAS significantly attenuated the expression of NLRP3 inflammasome and the intensity of SNL-induced mechanical allodynia. NLRP3 inflammasome inhibitor, MCC950, also attenuated the intensity of allodynia, but the effect is less strong and shorter than that of GAS.

**Conclusions:**

Expression of NLRP3 inflammasome and IL-1β is greatly increased and mostly found in the neurons at the spinal level in SNL model, and IT gastrodin exerts a significant anti-allodynic effect in SNL model partly through suppressing the expression of NLRP3 inflammasome.

**Supplementary Information:**

The online version contains supplementary material available at 10.1186/s12906-024-04519-w.

## Introduction

Neuropathic pain is caused by a lesion or disease affecting the somatosensory system in the peripheral or central nervous system [1]. Often refractory to current treatments, neuropathic pain underscores the lack of well-established mechanisms in its development and maintenance. A wealth of evidence implicates neuroinflammation, defined as the inflammatory response to various insults in the nervous system, as a pivotal player in the pathophysiology of neuropathic pain [2, 3].

Gastrodin (GAS) is a main bioactive component of Gastrodia elata Blume, an herbal plant that has traditionally been used in Chinese medicine. Its therapeutic effects have been demonstrated in CNS preclinical models of ischemic or neurodegenerative diseases in which the neuroinflammation plays an important role [4–6]. Several investigations have shown that the effect of GAS is related with the inhibition of inflammasomes [7–9], which are a group of cytosolic multimeric protein complexes that have a critical function in innate immune responses and neuroinflammation [10, 11].

The nucleotide-binding oligomerization domain (NOD)-like receptor protein 3 (NLRP3) inflammasome, which is the best characterized inflammasome, consists of an immune sensor protein (NLRP3), adaptor molecule (apoptosis-associated speck-like protein containing a caspase recruitment domain [ASC]), and downstream protease (pro-caspase-1). When NLRP3 is activated by endogenous or exogenous danger signals, the NLRP3 inflammasome is generated and caspase-1 is subsequently activated, leading to the conversion of pro-interleukin (IL)-1β to mature IL-1β [12–15].

Although the analgesic effects of GAS have been reported in human migraine and several rodent models of pain induced by peripheral inflammation, diabetes, chemotherapy, and peripheral nerve injury [16–20], the mechanisms underlying its action concerning neuroinflammation and the NLRP3 inflammasome at the spinal level remain to be elucidated. In addition, the extent to which inhibition of NLRP3 inflammasome contributes to the effect of GAS in the SNL model has not been explored.

The present study explored the role of NLRP3 inflammasome in the anti-allodynic effect of intrathecal (IT) GAS by examining the changes in NLRP3 inflammasome expression in the spinal cord and comparing the effect of IT treatment with GAS and NLRP3 inflammasome inhibitor in a rat model of L5/6 spinal nerve ligation (SNL).

## Materials and methods

### Experimental animals and implantation of intrathecal catheter

All experiments were performed in accordance with the International Association for the Study of Pain Guidelines for the Use of Animals in Research, and the experimental protocol was approved by the Institutional Animal Care and Use Committee [CNUH-IACUC-21,056]. The current study is a preclinical animal study and the results are described in accordance with the ARRIVE guideline.

Male Sprague–Dawley rats (7 weeks, 220–250 g; Damool Science, Daejeon, Korea) were acclimated to the laboratory environment for 3 days before use and given free access to a standard rat diet and tap water. The room temperature was maintained at 20–23 °C with an alternating 12 h light/dark cycle. In all procedures including catheter implantation, surgery for spinal nerve ligation and tissue sampling, the animals were anesthetized with the inhalation of 2–3 vol% of sevoflurane which was adjusted to provide optimal depth of anesthesia, delivered at a flow rate of 2 L/min using a mixture of air and oxygen.

Implantation of an IT catheter was performed as previously reported [21]. A polyethylene tube with an inner and outer diameter of 0.28 and 0.64 mm (PE-10 catheter; Becton, Dickinson, and Company, Franklin Lakes, NJ, USA) was used. One side of the PE-10 catheter was stretched to reduce the diameter before implantation. With the animals under general anesthesia with sevoflurane, the catheter was inserted into the IT space through the atlanto-occipital membrane and moved 8.5 cm in the caudal direction until it reached the lumbar enlargement. The other end of the PE-10 catheter was placed exteriorly on top of the animal’s head and plugged with a stainless steel wire to prevent the catheter from clogging and for later administration of experimental agents. Following implantation of the IT catheter, the animals were given a subcutaneous injection of 5 mL saline, and allowed to recover in individual cages for 5 days before further experiments.

Motor function was assessed by evaluating the righting reflex and place-stepping reflex daily. The pinnae and corneal reflexes were also examined to check for sensory deficits. Animals that showed neurological deficits after the implantation or thereafter were immediately sacrificed with an overdose of sevoflurane. In addition, after the experiment was completed, the lumbar spine of each animal was dissected to ensure that the IT catheter had been placed correctly in the IT space, and no animals had a misplaced IT catheter.

### Von Frey test for behavioral assessments of mechanical allodynia

The animals were given a 15 min acclimation period in a cage with a wire mesh floor. After this period, the allodynic responses to mechanical stimuli were measured using the von Frey test. Filaments with forces of 0.41 to 15.2 g were applied perpendicular to the middle of the plantar surface through the wire mesh floor. Each application was maintained for 5 s or until the occurrence of paw lifting or licking, which were considered positive responses. A filament with a force of 2 g was used first, and then the force was selected by the up-and-down paradigm to determine the paw withdrawal threshold (PWT) of 50% probability [22]. The behavioral test was performed by a researcher blinded to the previous treatment.

### Spinal nerve ligation for inducing mechanical allodynia

Mechanical allodynia was induced using a peripheral nerve injury model, L5/6 spinal nerve ligation (SNL) [23]. Five days after recovery from IT catheter implantation, the animals underwent a Sham operation or SNL. Each animal was placed in the prone position under general anesthesia with sevoflurane, and a longitudinal incision was made on the left side (about 3 cm in length and 5 mm lateral from the midline) at the L4–S2 levels. The left paraspinal muscles were separated from the spinous processes. The L6 transverse process was carefully removed with a small rongeur to visually identify the L4–L6 spinal nerves. The left L5 and L6 spinal nerves were isolated, and each nerve was tightly ligated with 6–0 silk thread in the SNL group, but not ligated in the Sham group. Following the SNL surgery, the rats with a baseline PWT < 10 g were excluded from the study before further experiment except day 1.

### Intrathecal administration of drugs

After confirming that the animals showed mechanical allodynia after surgical SNL, gastrodin (Sigma-Aldrich, St. Louis, MO, USA) or vehicle (saline) was intrathecally injected to investigate the effect of GAS on mechanical allodynia and expression of the NLRP3 inflammasome in SNL. In addition, the effect of MCC950 (InvivoGen, San Diego, CA, USA), a selective NLRP3 inflammasome inhibitor, was examined to compare its anti-allodynic effect with that of GAS. MCC950 was dissolved in dimethyl sulfoxide and then diluted in normal saline. The study dose was selected based on previous reports and the results of our preliminary test that had examined the maximum soluble dose for each drug with no side effects [19, 24].

Every IT administration of the experimental agent was followed by flushing the catheter with 10 µL vehicle. Behavioral study using von Frey test for mechanical allodynia started 10 min after the completion of IT drug administration. Removal of spinal cord and dorsal root ganglion for Western blotting was performed 2.5 h after IT injection of GAS. None of the animals showed side effects after being injected with the study doses of GAS or MCC950.

### Experiment protocol

**Fig. 1:**
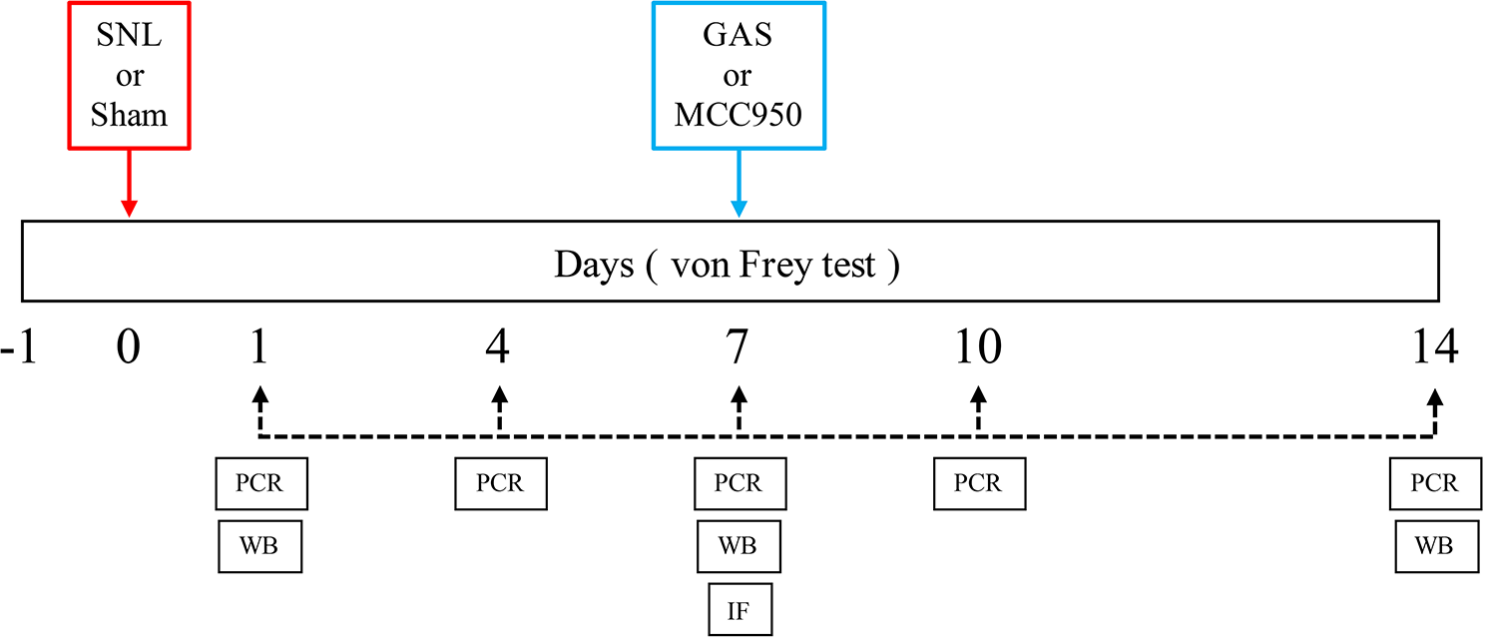
Schedule according to the IT treatment and sample collection. On day 0, SNL or Sham surgery was performed to establish the model. The dates for collection of spinal cord and dorsal root ganglion are as follows: (1) PCR: day 1, 4, 7, 10, and 14; (2) WB: day 1, 7, and 14; (3) IF: day 7. After finishing the experiments for changes of pain behavior and NLRP3 inflammasome, we started the second experiment in which animals were given the IT treatment with GAS, MCC950, or Vehicle on day 7 and its effect on pain behavior and NLRP3 inflammasome was investigated. GAS: gastrodin, IT: intrathecal, SNL: spinal nerve ligation, PCR: real-time polymerase chain reaction, WB: Western blotting, IF: immunofluorescence

Two sets of experiments were performed in separate group of animals; one is for changes of pain behavior and NLRP3 inflammasome expression in SNL, and the other for the effect of GAS treatment (Fig. 1). Experiment groups were divided according to surgery (SNL or Sham), IT treatment (vehicle, gastrodin, MCC950) or the days following the surgery. Animals were randomly assigned into one of the groups by generating a blocked allocation sequence using R (version 3.63) with equal allocation ratio. The primary researchers of this study identified the number of animals excluded from each group after the random assignment and assigned additional animals to maintain the same number in each group. The number of animals in each group was determined in reference with our experience and review of the previous experiments on MCC950 and gastrodin [17, 25].

In the first experiment, mechanical allodynia was measured using the von Frey test in two groups of animals (Sham and SNL) 1 day before and 1, 4, 7, 10, and 14 days after the surgery. We also collected the spinal cord and dorsal root ganglion (DRG) and examined the changes in NLRP3 inflammasome and IL-1β expression via real-time polymerase chain reaction (PCR) and Western blotting in separate groups of Sham and SNL animals. In addition, an immunofluorescence study on the spinal cord and DRG was performed in animals in the SNL group to locate the expression of the NLRP3 inflammasome including NLRP3, ASC, and caspase-1 on day 7 after SNL according to the PCR and Western blotting data.

In the second experiment, we tested the acute effect of IT administration of GAS (60, 300 and 600 µg) or MCC950 (3, 10, 30 and 100 µg) on mechanical allodynia on day 7 after SNL. Additional experiment was performed for examining the duration of anti-allodynic effect of both drugs. The effect of GAS (600 µg) on the expression of NLRP3 inflammasome-related protein was also examined using Western blotting of the spinal cord and DRG.

### Real-time PCR

After sacrificing each rat, the left dorsal quadrant of lumbar spinal cord enlargement and the left L5/6 DRG were quickly removed. Total RNA was extracted using Trizol reagent (Takara, Shiga, Japan), and 1 µg of total RNA was converted into complementary DNA (cDNA) using a PrimeScript RT reagent kit (Takara, Shiga, Japan). Then the synthesized cDNA samples were diluted and stored at − 80℃ until further experimentation. The cDNA was amplified using the specific primers listed as follows: 5′-agactggaaagagcttggcc-3′ (Nlrp3 forward primer), 5′-catccgcagccaatgaacag-3′ (Nlrp3 reverse primer), 5′-acagtaccaggcagttcgtg-3′ (Asc forward primer), 5′-ccaagtagggctgtgtttgc-3′ (Asc reverse primer), 5′-ccgagtggttccctcaagtt-3′ (Casp1 forward primer), 5′-tgctgcagataatgagggca-3′ (Casp1 reverse primer), 5′-tcctctgtgactcgtgggat-3′ (Il1b forward primer), 5′-tcacatgggtcagacagcac-3′ (Il1b reverse primer), 5′-atgaagatcctgaccgagcg-3′ (Actb forward primer), 5′-tgctcgaagtctagggcaac-3′ (Actb reverse primer). All the primers were synthesized by GenoTech (Daejeon, Korea). All PCR reactions were measured using TB Green Fast qPCR Mix (Takara, Shiga, Japan). PCR was performed in accordance with the manufacturer’s instructions. The expression of target mRNA was evaluated using the 2^−ΔΔCT^ method.

### Western blotting

The left dorsal quadrant of the lumbar spinal cord and the left L5/6 DRG were lysed in RIPA buffer containing a protease inhibitor and a phosphatase inhibitor. After 30 min of incubation, the lysates were centrifuged at 13,000 rpm for 15 min at 4 °C, and the supernatant was collected. Equal amounts of protein samples were separated by SDS/PAGE under denaturing conditions. Each protein sample obtained from one animal was run on three different gels. After transferring the proteins to a PVDF membrane, each membrane was horizontally cut according to the molecular weight of the target protein, using a protein marker as a reference. One of the three membranes was used for NLRP3 (118 kDa) and ASC (22 kDa), another for cleaved-caspase-1 (20 kDa) and caspase-1 (45 kDa), and the last for IL-1β (17 kDa).

The membranes were blocked in 5% skim milk or 5% bovine serum albumin for 1 h at room temperature and incubated with primary antibodies in a blocking buffer (skim milk or bovine serum albumin) overnight at 4 °C. The following primary antibodies were used according to the manufacturers’ instructions: NLRP3 (1:500; Novus Biologicals, Centennial, CO, USA), ASC (1:500; Novus Biologicals), caspase-1 (1:1000; Novus Biologicals), and IL-1β (1:500; Abcam, Cambridge, UK). The next day, after washing with Tris Buffered Saline-T, the membranes were incubated with secondary antibodies at room temperature for 2 h. Rabbit and mouse HRP- conjugated secondary antibodies (1:3000; Cell Signaling Technology, Danvers, MA, USA) were used. β-tubulin (1:1000; Santa Cruz Biotechnology, Dallas, TX, USA) was used as a loading control to obtain the relative intensity of target proteins. In the case of caspase-1, the relative intensity of cleaved-caspase-1 to pro-caspase-1 was determined.

### Immunofluorescence

The rats were deeply anesthetized with sevoflurane and transcardially perfused with 0.9% sterilized saline followed by 4% paraformaldehyde in 0.1 M phosphate buffer (pH 7.4). The lumbar enlargement of the spinal cord and left L5/6 DRG were dissected out and post-fixed in 4% paraformaldehyde at 4 °C overnight. Then the tissues were immersed in 30% sucrose in 0.1 M phosphate buffer until the tissues sank. The tissues were frozen in OCT compound on dry ice and then sectioned transversely on a cryostat at 15 μm thickness for the spinal cord and 10 μm thickness for the DRG.

The sections were blocked with 3% (v/v) normal Donkey serum containing 0.3% Triton-X100 for 60 min at room temperature and then incubated overnight at 4 °C with the following primary antibodies: rabbit anti-NLRP3 polyclonal antibody (1:50, Novus Biologicals, Centennial, CO, USA), rabbit anti-ASC polyclonal antibody (1:100, Novus Biologicals) or rabbit anti-caspase-1 polyclonal antibody (1:50, Novus Biologicals). Antibody binding to tissue sections was visualized after incubation with Donkey anti-rabbit IgG conjugated to Alexa Fluor 488 (1:500; Jackson ImmunoResearch) for 2 h at room temperature. The sections were rinsed in 0.01 M phosphate-buffered saline, and cover slips were applied.

For double-label immunofluorescence evaluation, spinal cord sections were incubated in 3% (v/v) normal Donkey serum for 30 min at room temperature and subsequently with rabbit anti-NLRP3 polyclonal antibody (1:100, Novus Biologicals), rabbit anti-ASC polyclonal antibody (1:100, Novus Biologicals) or rabbit anti-caspase-1 polyclonal antibody (1:50, Novus Biologicals) at 4 °C overnight. Antibody binding to tissue sections was visualized after incubation with Donkey anti-rabbit IgG conjugated to Alexa Fluor 488 (1:500; Jackson ImmunoResearch, West Grove, PA, USA) for 2 h at room temperature. Then, the tissue sections were incubated with the following antibodies: goat anti-Iba-1 monoclonal antibody (1:1000, Abcam, Cambridge, UK), mouse anti-NeuN monoclonal antibody (1:1,000, Millipore, Burlington, MA, USA) or mouse anti-GFAP polyclonal antibody (1:200, Santa Cruz, Dallas, TX, USA) for 3 h at room temperature. Antibody-binding to tissue sections was visualized after incubating with Donkey anti-mouse IgG conjugated to Alexa Fluor 594 (1:500; Jackson ImmunoResearch) or Donkey anti-goat IgG conjugated to Alexa Fluor 594 (1:500; Jackson ImmunoResearch) for 1 h at room temperature.

Immunofluorescent images were obtained using a fluorescence microscope (EVOS M5000; Thermo Fisher Scientific, Waltham, MA, USA) at 200X. The percentage of the stained target area was examined to quantify and compare the fluorescent intensity and immunoreactivity of the sectioned spinal cord and DRG. Immunofluorescent image analysis was performed using Image J.

### Statistical analysis

Data obtained from the von Frey test are presented as mean ± standard deviation. The intensity of mechanical allodynia was converted into the area under the curve (AUC), which was obtained from the time-course curve of the PWT using the trapezoidal rule. A smaller AUC indicated that the animals showed a more intense allodynic response than those with a larger AUC. Data for Western blotting, real-time PCR, and immunofluorescence are presented as mean ± standard error of the mean.

A total of 231 rats underwent the SNL or Sham surgery, and 15 animals with SNL surgery did not develop the state of mechanical allodynia. Of 231 animals, 152 rats were received the i.t catheter implantation and 16 were excluded due to the motor impairment. In total, data of 200 animals were analyzed and presented in the results.

All data were analyzed using SPSS software ver. 22.0 (IBM Corp., Armonk, NY, USA). Normality of the data was tested with Shapiro-Wilk test. In the current study, the differences between the groups were analyzed by independent t-test or one-way analysis of variance followed by Tukey’s *post hoc* comparison test. A p-value < 0.05 was considered significant.

## Results

### Induction of mechanical allodynia in spinal nerve ligation model

**Fig. 2:**
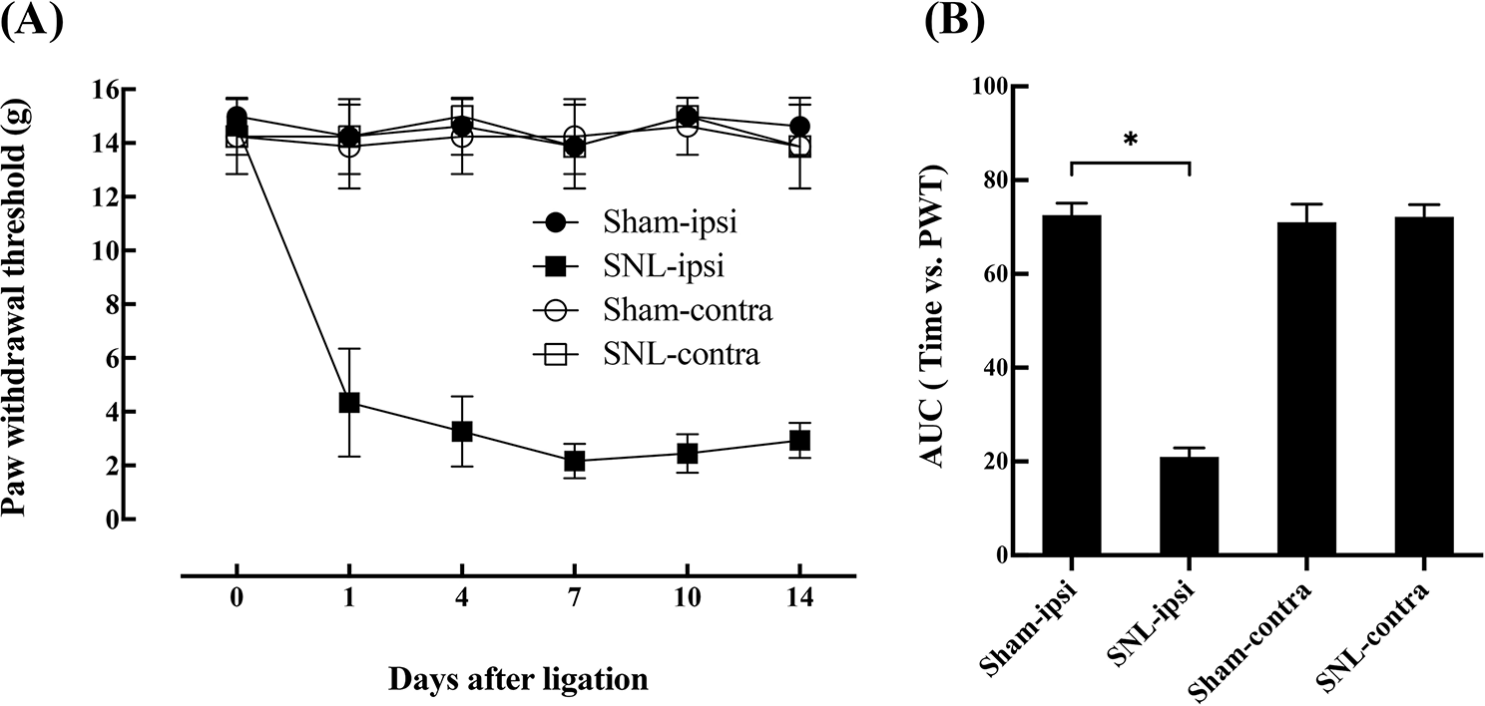
Time course of the PWT (**A**) and the corresponding AUC (**B**) of ipsilateral and contralateral hind paw after SNL or Sham surgery. L5/6 SNL significantly decreased the PWT in the ipsilateral side of the SNL group compared to the other groups. *P < 0.05; *n* = 8 in each group; error bars indicate mean ± standard deviation. SNL: spinal nerve ligation, PWT: paw withdrawal threshold, AUC: area under the curve, ipsi: ipsilateral, contra: contralateral

Animals in the SNL group showed a significant decrease in the PWT of the ipsilateral hind paw from 1 to 14 days after ligation of the L5 and L6 spinal nerve (Fig. 2). No significant changes in the Sham group or the contralateral paw in the SNL group were observed.

### Increase in NLRP3 inflammasome expression in neurons in spinal dorsal horn and DRG in the SNL model

**Fig. 3:**
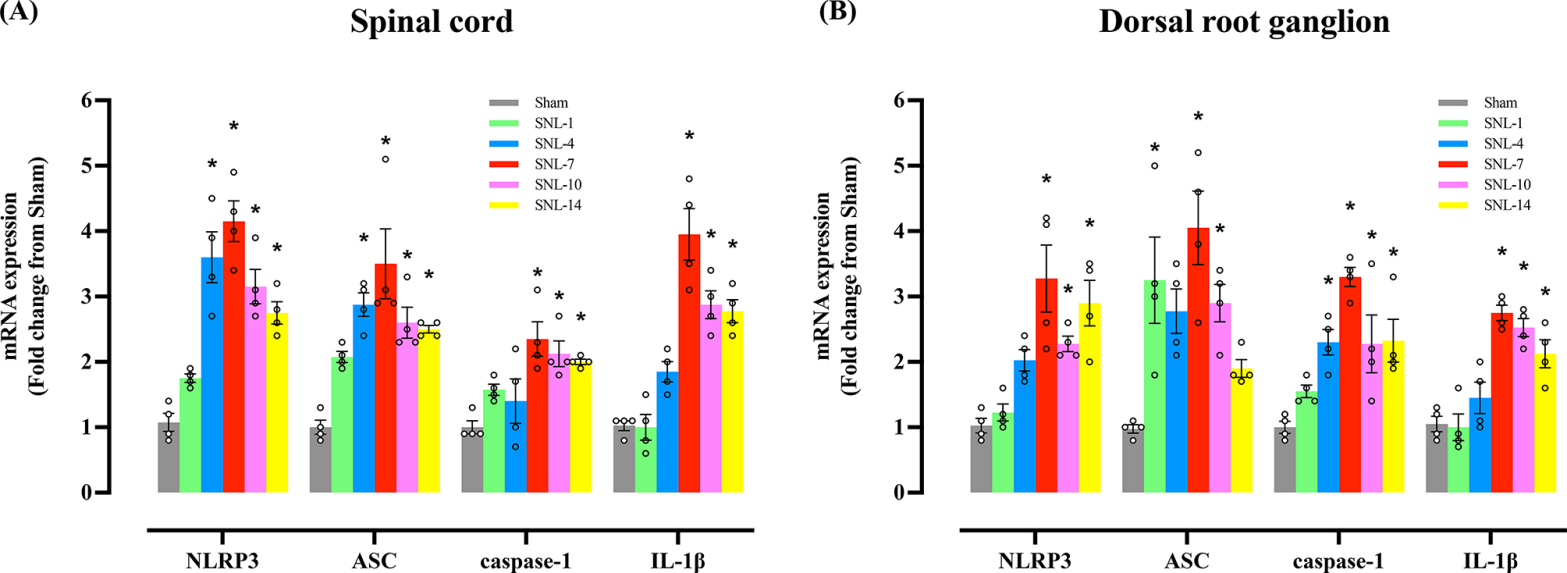
Changes in mRNA expression level of NLRP3 inflammasome components and IL-1β in spinal cord (**A**) and DRG (**B**) measured via real-time PCR. Measurement was taken on day 1, 4, 7, 10, and 14 after L5/6 SNL. The fold change from the Sham group peaked on day 7 for all the NLRP3 inflammasome components and IL-1β mRNA. *P < 0.05 vs. Sham group. *N* = 4 in each group and the error bars indicate mean ± standard error. Circles in the graph represents the values of individual sample in each group. SNL: spinal nerve ligation, DRG: dorsal root ganglion

In real-time PCR, mRNA expression of NLRP3, ASC, caspase-1, and IL-1β was significantly increased in the spinal cord and DRG after SNL (Fig. 3). The increases started on day 4 or 7 and continued until day 14. The upregulation peaked on day 7 with the changes from Sham being more than two- to four-fold.

**Fig. 4:**
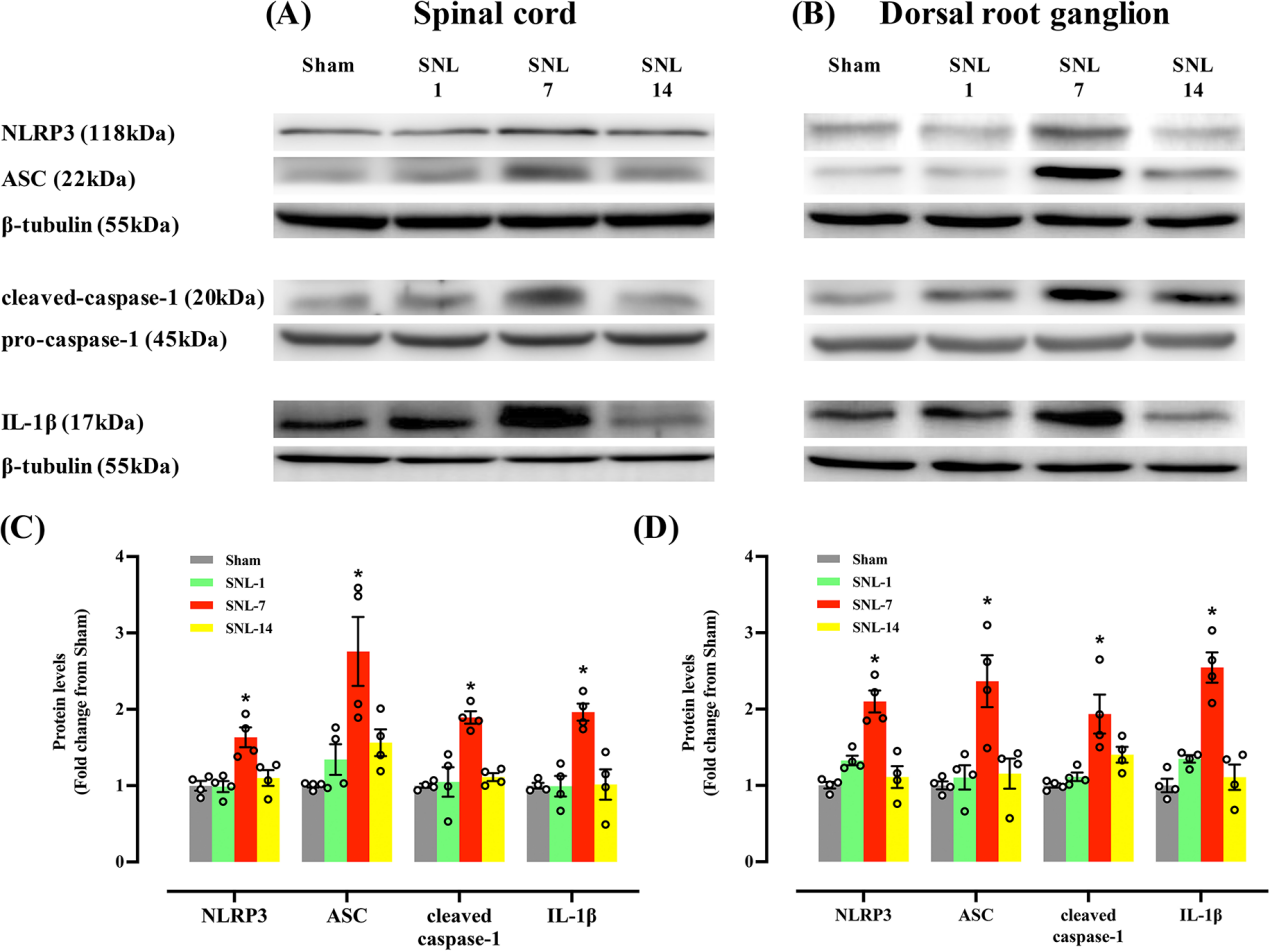
Western blotting was performed to examine the changes in the protein level of NLRP3 inflammasome components and IL-1β in ipsilateral dorsal quadrant of lumbar spinal cord spinal cord (**A**, **C**) and dorsal root ganglion (**B**, **D**). Animals underwent Sham or L5/6 SNL surgery and were grouped per surgery or postoperative days (days 1, 7, and 14). Significant increases in NLRP3, ASC, cleaved-caspase-1, and IL-1β were observed in the SNL-7 group compared to the Sham group. The above blots were derived from three distinct gels/membranes, with each membrane horizontally sectioned according to a molecular weight marker for both the target protein and its loading control: (1) NLRP3 and ASC (β-tubulin), (2) cleaved-caspase-1 (pro-caspase-1), (3) IL-1β (β-tubulin). *P < 0.05 vs. Sham, SNL-1, or SNL-14 groups. *N* = 4 in each group and the error bars indicate mean ± standard error of the mean. Circles in the graph represents the values of individual sample in each group. SNL: spinal nerve ligation. Original images with edges and additional replicates are shown in the Supplementary Material 1 and 2 (supplementary Fig. 4- additional replicates and supplementary Fig. 4- edges)

The changes in the protein level of NLRP3 inflammasome was observed to vary according to the days after SNL surgery. Additionally, the protein levels of NLRP3, ASC, cleaved caspase-1 (p20), and mature IL-1β (p17) were significantly higher in the SNL group than in the Sham group in the spinal cord and DRG on day 7, but not on day 1 or 14 (Fig. 4). Although the peak increase in protein level is observed on day 7, similar to the mRNA level, the changes were not parallel to the time course of the paw withdrawal threshold which is significantly decreased throughout the study period.

**Fig. 5:**
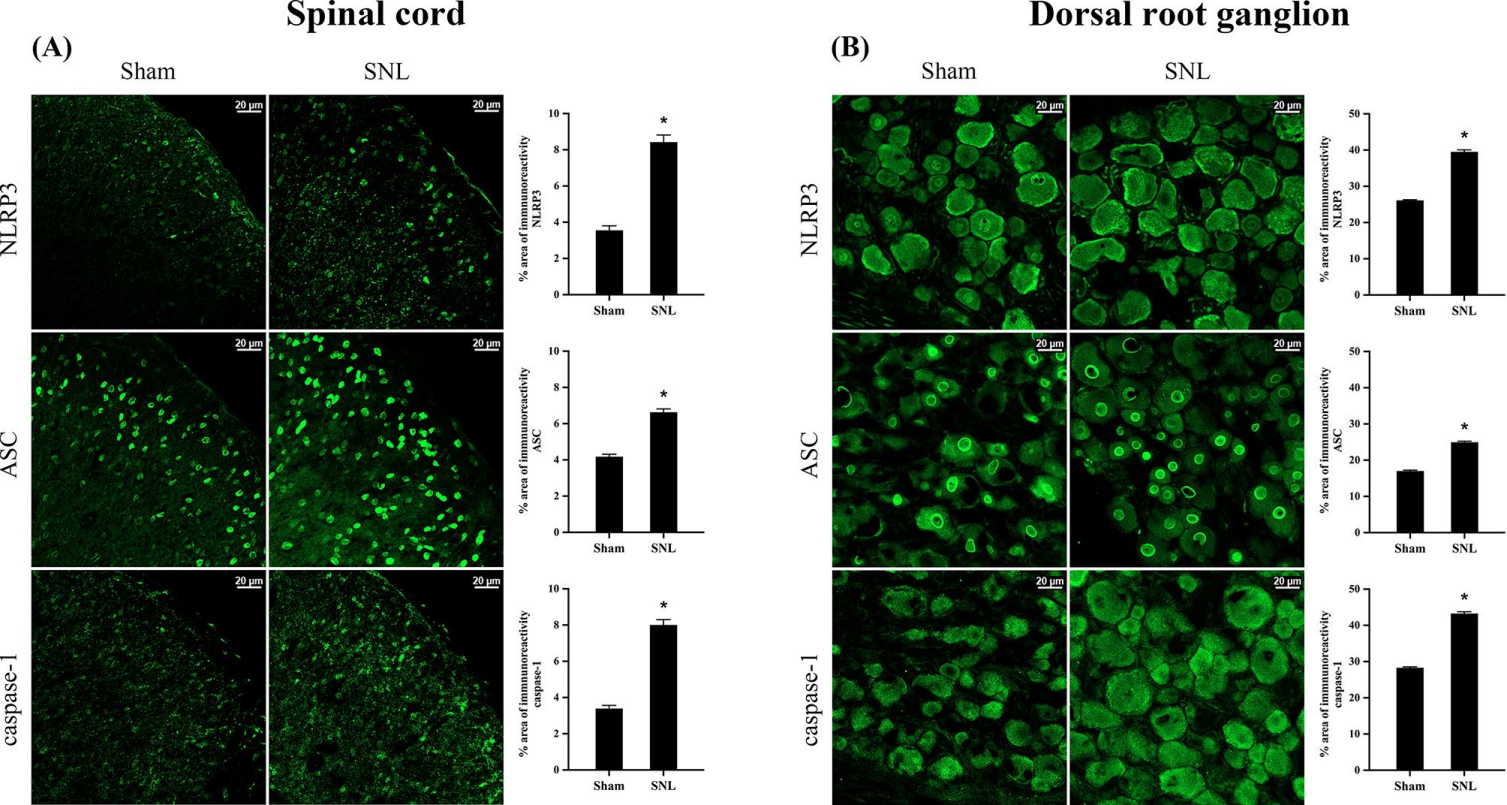
Increase of NLRP3 inflammasome expression 7 days after L5/6 SNL. The images represent the fluorescence intensity obtained in each group and the difference between Sham and SNL group was analyzed using the percent area with positive fluorescent activity of NLRP3, ASC, and caspase-1. The area for all the NLRP3 components increases significantly in SNL animals compared to Sham group. *P < 0.05. *N* = 4 in each group and the error bars indicate mean ± standard error of the mean. Scale bar: 20 μm. Iba-1: microglia, GFAP: astrocytes, NeuN: neuron, SNL: spinal nerve ligation, DRG: dorsal root ganglion. Images from the contralateral dorsal horn are shown in the Supplementary Material 3 (supplementary Fig. 5-contralateral)

Consistent with the Western blotting, SNL group showed a significant increase in the percent area of (+) fluorescent intensity of NLRP3, ASC, and caspase-1 compared to that of Sham group on day 7 after Sham or SNL surgery (Fig. 5).

### Cellular expression of NLRP3 inflammasome in spinal dorsal horn and DRG

**Fig. 6:**
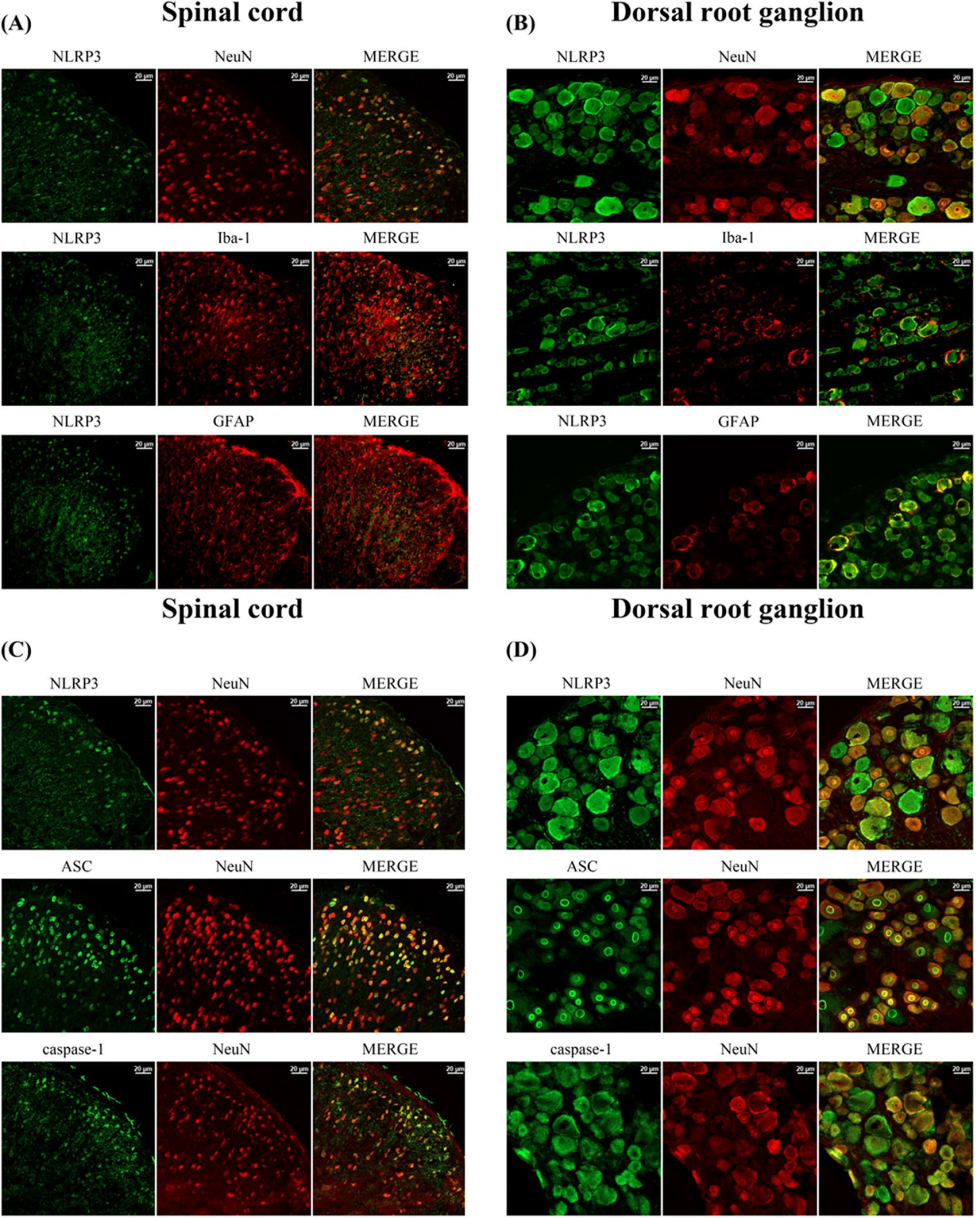
Difference in cellular expression of NLRP3 inflammasome 7 days after L5/6 SNL. Double immunofluorescence study shows that NLRP3 is predominantly colocalized with NeuN in the left dorsal horn of the lumbar spinal cord (**A**) and left L5/L6 DRG (**B**). Only a small amount of NLRP3 is colocalized with GFAP in the DRG, and NLRP3 was rarely colocalized with Iba-1 in dorsal horn and DRG. Additional staining with other sections for ASC and caspase-1 revealed a similar pattern to NLRP3. Expression of ASC and caspase-1 was also observed with NeuN in the left dorsal horn of the lumbar spinal cord (**C**) and left L5/6 DRG (**D**). *N* = 4 in each group. Scale bar: 20 μm. Iba-1: microglia, GFAP: astrocytes, NeuN: neuron, SNL: spinal nerve ligation, DRG: dorsal root ganglion. Images from the contralateral dorsal horn are shown in the Supplementary Material 4 (supplementary Fig. 6AC-contralateral)

Differential cellular expression of NLRP3 was observed at the spinal level. In double immunofluorescence analyses, the expression of NLRP3 predominantly colocalized with NeuN, while showing minimal colocalization with GFAP or Iba-1 in the spinal dorsal horn (Fig. 6A). A similar pattern was also observed in the DRG, where NLRP3 colocalized mainly with NeuN, although a small amount of fluorescent intensity was also detected with GFAP (Fig. 6B). In addition, ASC and caspase-1 expression were also colocalized with NeuN in the spinal dorsal horn and DRG (Fig. 6C and D).

### Gastrodin and MCC950 relieves SNL-induced mechanical allodynia with different efficacy

**Fig. 7:**
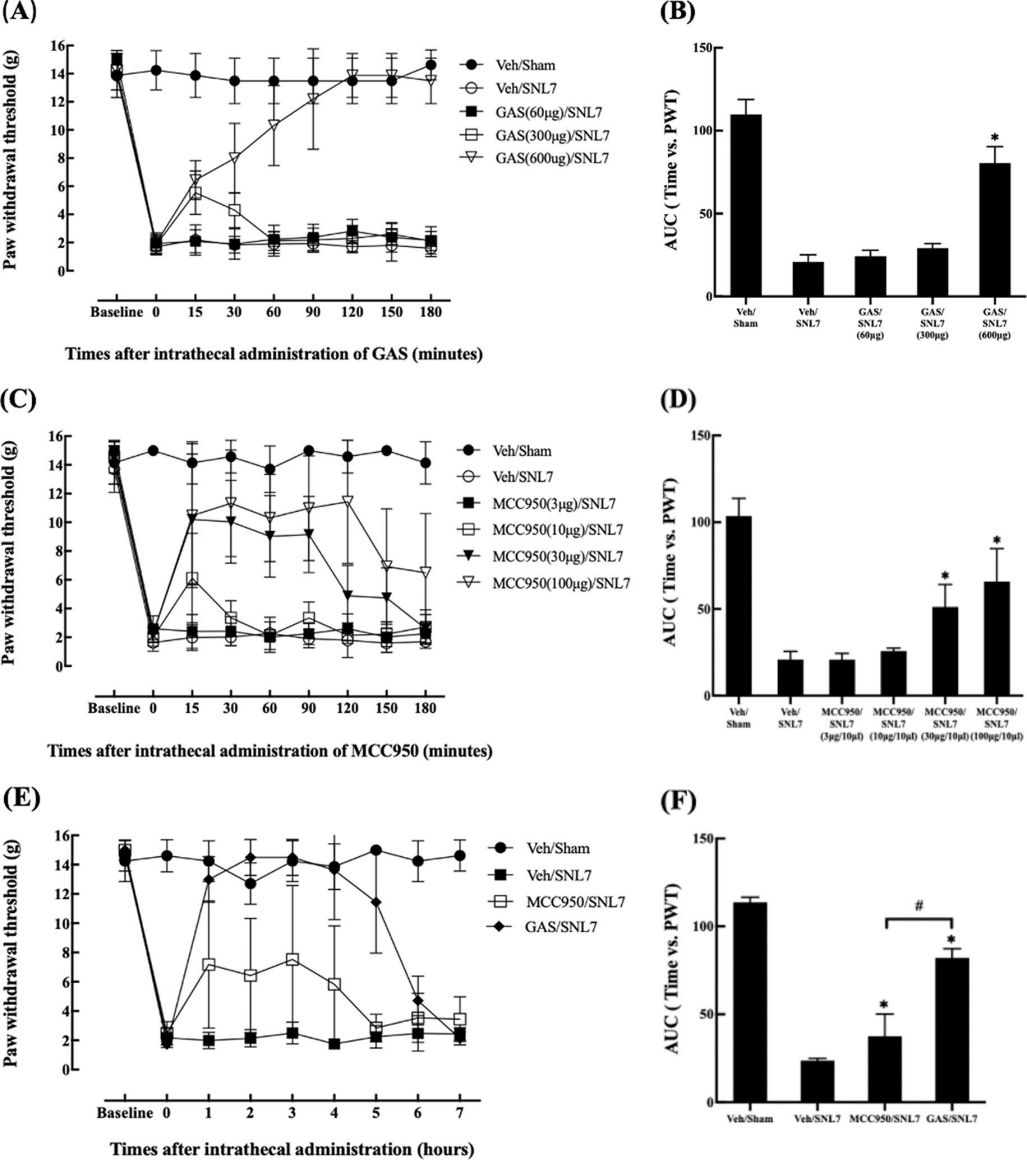
Effect of IT treatment of GAS or MCC950 on the intensity of mechanical allodynia on day 7 after L5/6 SNL. The time course of the PWT after IT injection of GAS and MCC950 is shown in the left panel, and the corresponding AUC is shown in the right panel. GAS (600 µg) produced a significant anti-allodynic effect with its maximal inhibitory effect being close to that of the Veh/Sham group (**A**, **B**). MCC950 also significantly increased the PWT, but its AUC was 63.6% (30 µg) and 87.3% (100 µg) of that of GAS (**C**, **D**). In additional study for comparing the two drugs for extended hours, a stronger and longer effect of GAS than MCC950 was observed (E, F). In the X-axis, baseline represents the PWT before SNL or Sham surgery, and 0 represents before IT injection of experiment drugs. *P < 0.05 vs. Veh/SNL7 group, #*P* < 0.05. *N* = 8 in each group and error bars indicate mean ± standard deviation. Veh: vehicle, GAS: gastrodin, IT: intrathecal, PWT: paw withdrawal threshold, SNL: spinal nerve ligation, DRG: dorsal root ganglion

A behavioral study on the effect of IT gastrodin (GAS) or MCC950 was performed on day 7, when the NLRP3 inflammasome-related components were most upregulated in PCR and Western blotting tests. Mechanical allodynia was measured using the von Frey test from 15 to 180 min after treatment with GAS or MCC950 (Fig. 7A-D). No significant changes in PWT were observed in the contralateral side with GAS or MCC950 in Sham or SNL animals (Supplementary Material 5; supplementary Fig. 7-von Frey-contralateral). However, both drugs significantly attenuated the SNL-induced mechanical allodynia in the ipsilateral side. Nevertheless, the AUC of MCC950 is smaller than of that of GAS, suggesting that the anti-allodynic effect of GAS is stronger than MCC950. Moreover, the onset and duration of the effect appeared to differ, as shown in the time course of PWT after IT treatment. However, as for GAS, the effect was not produced at a dose below 600 µg and the treatment with the dose higher than 600 µg caused side effects including abnormal behavior.

Additional study was performed to directly compare the anti-allodynic effect between the two drugs (Fig. 7E and F). The AUC for GAS (600 µg) is significantly larger than that of MCC950 (100 µg), indicating the anti-allodynic effect of GAS is stronger and longer-lasting than that of MCC950. The effect of GAS manifested 1-hour post-treatment and persisted for up to 5 h, whereas the effect duration of MCC950 was only approximately 2 h.

### Intrathecal gastrodin suppresses upregulation of the NLRP3 inflammasome and IL-1β

**Fig. 8:**
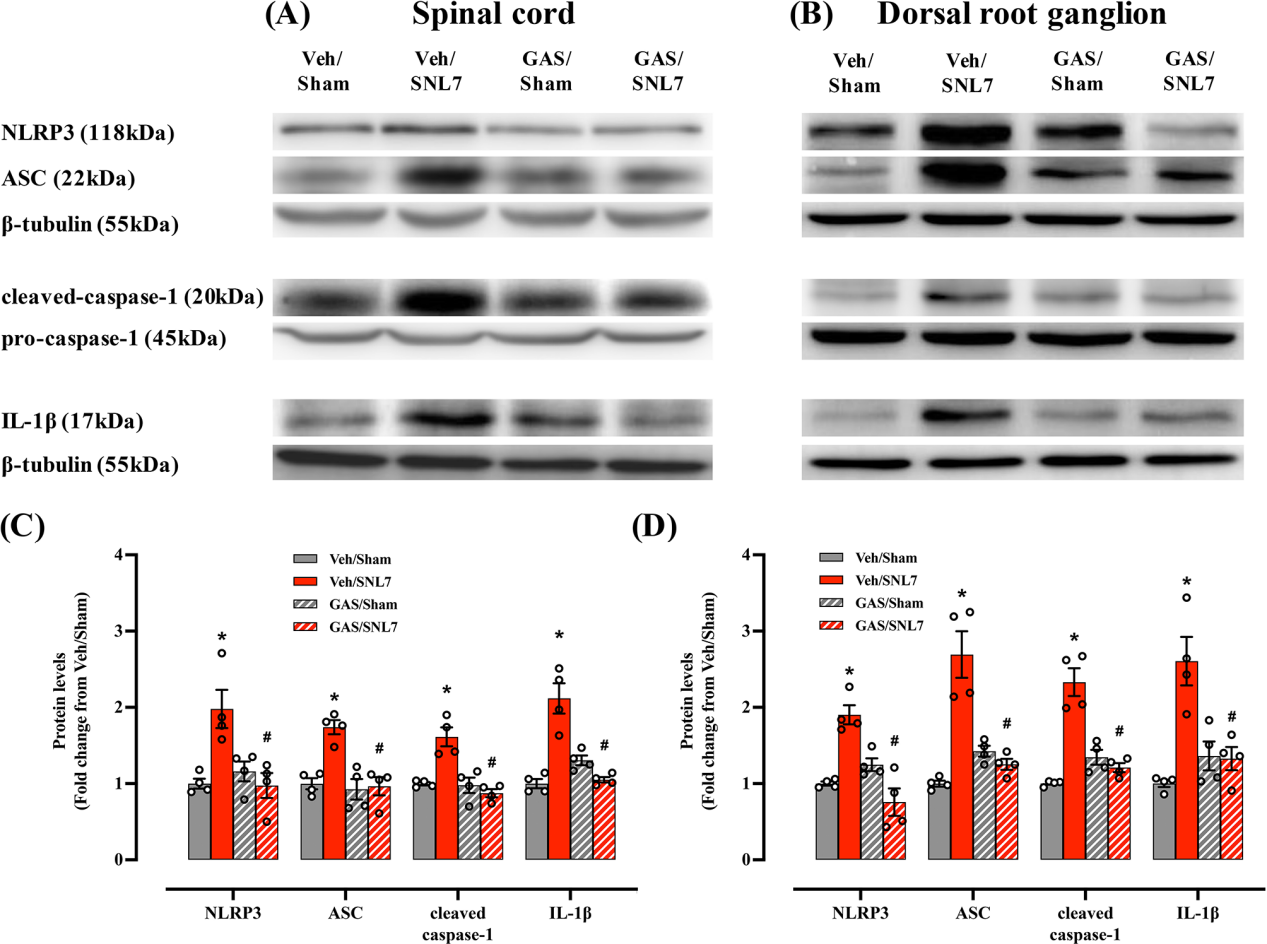
Effects of IT GAS on SNL-induced upregulation of NLPR3, ASC, caspase-1, and IL-1β assessed by Western blotting of (**A**) spinal cord (ipsilateral dorsal quadrant) and (**B**) DRG. Tissues were harvested 2.5 h after IT injection of GAS or vehicle. (**C**, **D**) Upregulated NLPR3, ASC, cleaved caspase-1, and IL-1β protein levels were decreased by IT GAS (600 µg /10 µL). There was no significant change in the Sham group after the injection of GAS (600 µg). The above blots were derived from three distinct gels/membranes, with each membrane horizontally sectioned according to a molecular weight marker for both the target protein and its loading control: (1) NLRP3 and ASC (β-tubulin), (2) cleaved-caspase-1 (pro-caspase-1), (3) IL-1β (β-tubulin). *P < 0.05 vs. Veh/Sham group; #*P* < 0.05 vs. Veh/SNL7 group. *N* = 4 in each group and the error bars indicate mean ± standard error. Circles in the graph represents the values of individual sample in each group. Veh: vehicle, GAS: gastrodin, IT: intrathecal, SNL: spinal nerve ligation, DRG: dorsal root ganglion. Original images with edges and additional replicates are shown in the Supplementary Material 6 and 7 (supplementary Fig. 8-additional replicates and supplementary Fig. 8-edges)

The effects of IT GAS on the protein expression of NLRP3 inflammasome-related components and IL-1β were examined on day 7 after SNL (Fig. 8). No significant effect was observed following IT GAS injection in Sham animals, as indicated by the comparison of NLRP3 inflammasome levels between Veh/Sham and GAS/Sham animals. However, IT treatment with GAS (600 µg) significantly reversed the upregulation of NLRP3 inflammasome-related components in SNL animals to levels comparable to those observed in Sham animals.

## Discussion

The present study showed that the NLRP3 inflammasome was significantly upregulated and predominantly expressed in neurons in the spinal cord dorsal horn and DRG in SNL model of rats. Treatment with IT GAS effectively reduced the upregulation of the NLRP3 inflammasome, leading to a reduction in the expression of IL-1β. Consistently, the mechanical allodynia was significantly attenuated by IT treatment with GAS. Although the NLRP3 inflammasome inhibitor, MCC950, also demonstrated a reduction in the intensity of mechanical allodynia, the anti-allodynic effect of IT GAS was stronger and more enduring than that of IT MCC950.

GAS is isolated from the rhizome of *Gastrodia elata*, an herbal plant used in Chinese traditional medicine. Its beneficial effects have been demonstrated in various preclinical models of central nervous system disorders, including Parkinson’s disease, dementia, depression, and cerebral ischemia [4]. Its analgesic effects have been explored in clinical trials, where a meta-analysis suggested its efficacy and safety in treating migraine headaches [20]. In addition, a multicenter study on the use of GAS for medication overuse-related headache was recently started [26]. By contrast, only a few studies have evaluated other types of pain using rodent model of inflammatory or neuropathic pain. For inflammatory pain induced by complete Freund’s adjuvant, intraperitoneal injection of GAS showed an antinociceptive effect by reducing the upregulation of glutamate receptors and glial activation in the anterior cingulate cortex [27]. At the spinal level, GAS inhibited spinal synaptic potentiation between primary afferent fibers and spinal lamina I neurons in models of inflammatory pain induced by bee venom and complete Freud’s adjuvant [19]. In terms of neuropathic pain, systemic administration of GAS relieved hyperalgesia and allodynia in models of painful diabetic neuropathy and chemotherapy-induced neuropathic pain via inhibition of DRG neuron hyperexcitability, inhibition of microglial activation, or reduction of TNF-α and IL-1β [16, 18]. However, investigations into the effects of GAS at the spinal level in neuropathic pain models, other than peripheral inflammatory pain, are scarce. In the current study, we administered GAS directly into the IT space and used a peripheral nerve injury-induced neuropathic pain model to examine the effect of GAS and the involvement of NLRP3 inflammasome at spinal level.

Activation of the NLRP3 inflammasome has been demonstrated in some neuropathic pain models, including chronic constriction injury of the sciatic nerve, spared nerve injury, and paclitaxel-induced neuropathic pain [28–31]. By contrast, a study using spared nerve injury model showed that NLRP3 knock out failed to reduce the intensity of hyperalgesia and allodynia suggesting no significant role of the NLRP3 inflammasome [32]. In that study, however, the levels of ASC, caspase-1, and IL-1β in mice with spared nerve injury did not differ between NLRP3^−/−^ and wild-type mice, which cannot be solely explained by the knockdown of NLRP3. In addition, two other reports using spared nerve injury of rats demonstrated a significant increase in the expression of the NLRP3 inflammasome as well as reduction of allodynia following intraperitoneal injection of MCC950, an NLRP3 inflammasome inhibitor [30, 33].

Previous studies have highlighted the critical role of neuronal apoptosis involving caspases in neuropathic pain following peripheral nerve injury, and its inhibition has shown promise as a therapeutic strategy [34, 35]. The current study further demonstrated a significant increase in the expression of ASC and caspase-1 in the spinal cord dorsal horn and DRG, indicating the activation of apoptotic pathway, which was attenuated by IT treatment of GAS. While the role of the NLRP3 inflammasome in neuropathic pain requires further clarification, some research suggests that GAS can modulate the activity of the NLRP3 inflammasome. Inhibition of the NLRP3 inflammasome by GAS has been demonstrated in vivo and in vitro using animal models of myocardial ischemia, cerebral ischemia, fulminant hepatitis, traumatic brain injury, septic shock, and cognitive dysfunction [7–9, 36–38]. Consistent with these findings, the current study also showed a strong suppression of NLRP3 inflammasome at spinal level and a corresponding anti-allodynic effect following IT administration of GAS.

An anti-allodynic effect of MCC950 was also observed in the current study, but the effect was less strong and shorter in duration than that of GAS. This difference in the anti-allodynic effect between GAS and MCC950 suggests that mechanisms other than NLRP3 inflammasome activation can be involved in the occurrence or maintenance of SNL-induced neuropathic pain. Furthermore, the increase in NLRP3 inflammasome expression in the SNL group was nearly eliminated to a level similar to that of the Sham group (Fig. 8). This suggests that there may be no significant difference in the effectiveness of suppressing NLRP3 expression between GAS and MCC950. Previous investigations also showed that GAS could have had other effects in the spinal cord, such as reduction of synaptic potentiation (neuronal hyperexcitability), inhibition of microglial activation, or proinflammatory cytokine production including TNF-α and IL-1β [16, 18, 19]. Given that neuropathic pain has a variety of causes and underlying mechanisms and GAS has multiple therapeutic effects in the nervous system, inhibition of NLRP3 inflammasome with GAS could be a useful strategy for treatment of neuropathic pain.

Immunofluorescence staining in the present study revealed that NLRP3, ASC, and caspase-1 were predominantly expressed in neurons in the spinal cord and DRG but scarcely in astrocytes of the DRG. However, previous investigations have reported conflicting results regarding cellular expression of the NLRP3 inflammasome [29, 30, 39–41]. It can be assumed that these various results are due to many differences among the studies, including the type of insult inducing an inflammatory response, species of rodent, site of expression and activation, or duration following insult in the nervous system. In the peripheral nerve injury model, neuron in the spinal cord might be the main cell which is involved in NLRP3 expression and activation, considering the direct mechanical damage to the nerves in the periphery as shown in the present study. However, NLRP3 expression was also observed in microglia, astrocytes, and neurons in the spinal cord, even in the same model such as chronic constriction injury [17, 25]. These results are far short of drawing a definite conclusion regarding cellular expression of the NLRP3 inflammasome at the spinal level. However, those results indicate that complex interactions occur continuously among various components, including neurons and glial cells, in the peripheral and central nervous systems in neuropathic pain.

One of several limitations of the present study is that the changes in NLRP3 inflammasome expression by MCC950 were not confirmed although the blockade of NLRP3 inflammasome activation using MCC950 in the spinal cord has been proved in previous studies [24, 25, 42, 43]. In addition, we did not examine the change in glial activation, which is one of the most important features of neuropathic pain. To reveal the mechanisms underlying the effect of GAS, it would be helpful to compare the difference in activation of microglia or astrocytes between GAS and MCC950 treatment. Another limitation would be that the anti-allodynic effect of GAS was tested with a single IT administration. Although GAS reduced the allodynia close to that of the Sham animals at the highest dose, chronic treatment with repeated injections with a lower dose could have ameliorated the pain behavior considering the chronicity of the neuroinflammatory response.

## Conclusions

In our study, IT administration of GAS demonstrates a potent anti-allodynic effect in the L5/6 SNL model, surpassing that of the NLRP3 inflammasome inhibitor. This result reflects the activity of GAS in modulating the neuroinflammation in the nervous system. Moreover, IT gastrodin abolishes the increase in the expression of NLRP3 inflammasome components including NLRP3, ASC, and caspase-1, resulting in the blockade of IL-1β production. These findings suggest that GAS exhibits a robust inhibitory activity against the neuroinflammatory responses associated with neuropathic pain, beyond NLRP3 inflammasome inhibition alone. Altogether, these results indicate that GAS acts through multiple mechanisms, not limited to the inhibition of NLRP3 inflammasome, at the spinal level and could serve as a potential drug candidate for the treatment of neuropathic pain.

### Electronic supplementary material

Below is the link to the electronic supplementary material.

Supplementary Material 1

Supplementary Material 2

Supplementary Material 3

Supplementary Material 4

Supplementary Material 5

Supplementary Material 6

Supplementary Material 7

## Data Availability

The datasets used and/or analyzed during the current study are available from the corresponding author on reasonable request.

## Abbreviations

GAS: gastrodin
NLRP3: nucleotide-binding oligomerization domain (NOD)-like receptor protein 3
ASC: apoptosis-associated speck-like protein containing a caspase recruitment domain
IL-1β: interleukin-1β
SNL: L5/6 spinal nerve ligation
DRG: dorsal root ganglion
IT: intrathecal
PWT: paw withdrawal threshold
