# Supplementary material for: Intrathecal gastrodin alleviates allodynia in a rat spinal nerve ligation model through NLRP3 inflammasome inhibition
Source: BMC Complement Med Ther. 2024 Jun 4;24:213. doi: 10.1186/s12906-024-04519-w (PMC11149323; doi:10.1186/s12906-024-04519-w)
Supplement: Supplementary file 1 — Supplementary Material 1 [file 12906_2024_4519_MOESM1_ESM.docx]

**
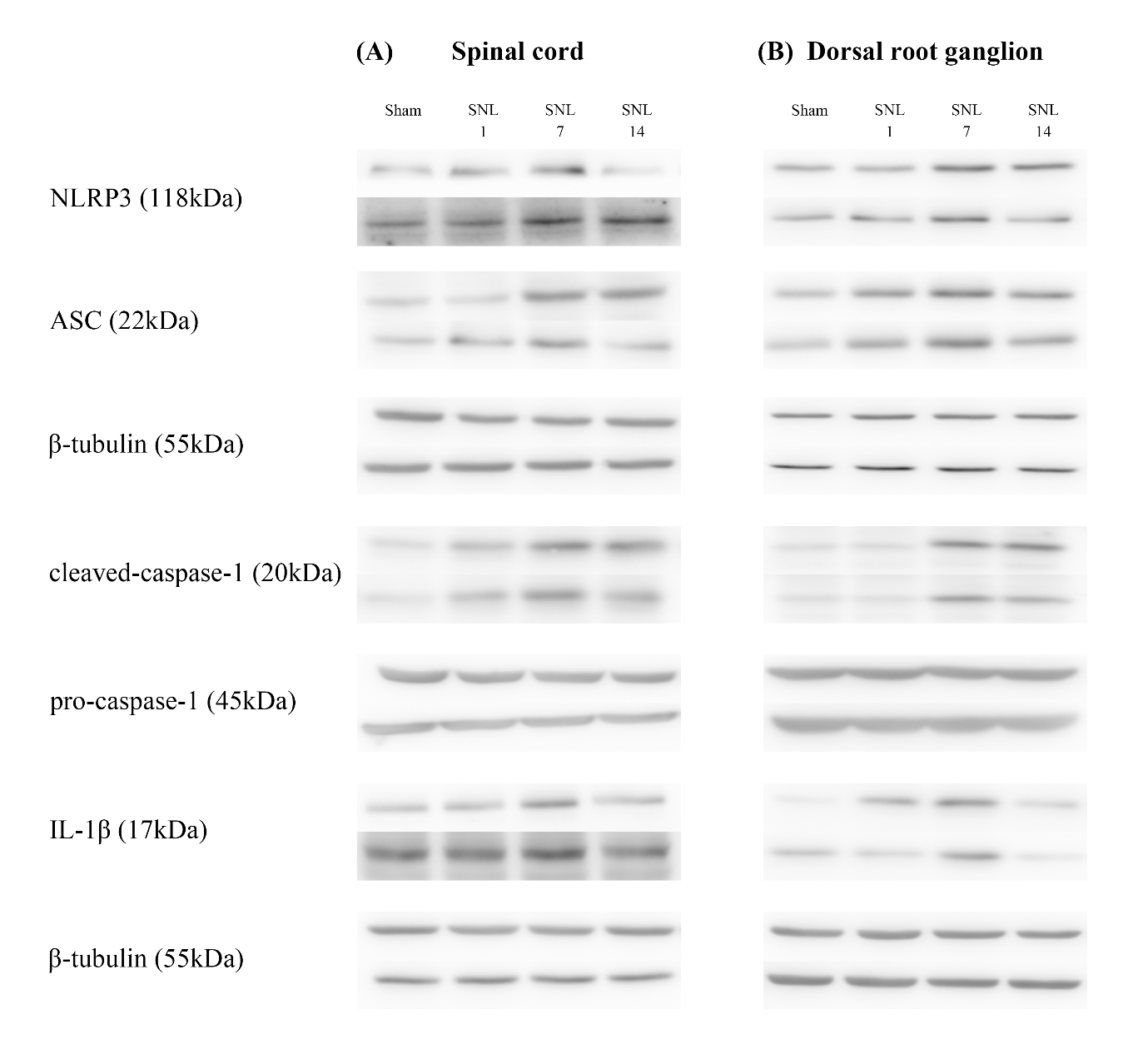
**

**Supplementary figure 4-additional replicates:** Images featuring replicates of the bands shown in Figure 4. Bands representing NLRP3 and ASC, cleaved-caspase-1, and IL-1beta, along with their respective loading controls, are displayed. These replicates were obtained from the same animal as depicted in Figure 4, but each band was cropped from different gel/membranes.
