# Supplementary material for: Intrathecal gastrodin alleviates allodynia in a rat spinal nerve ligation model through NLRP3 inflammasome inhibition
Source: BMC Complement Med Ther. 2024 Jun 4;24:213. doi: 10.1186/s12906-024-04519-w (PMC11149323; doi:10.1186/s12906-024-04519-w)
Supplement: Supplementary file 2 — Supplementary Material 2 [file 12906_2024_4519_MOESM2_ESM.docx]

**
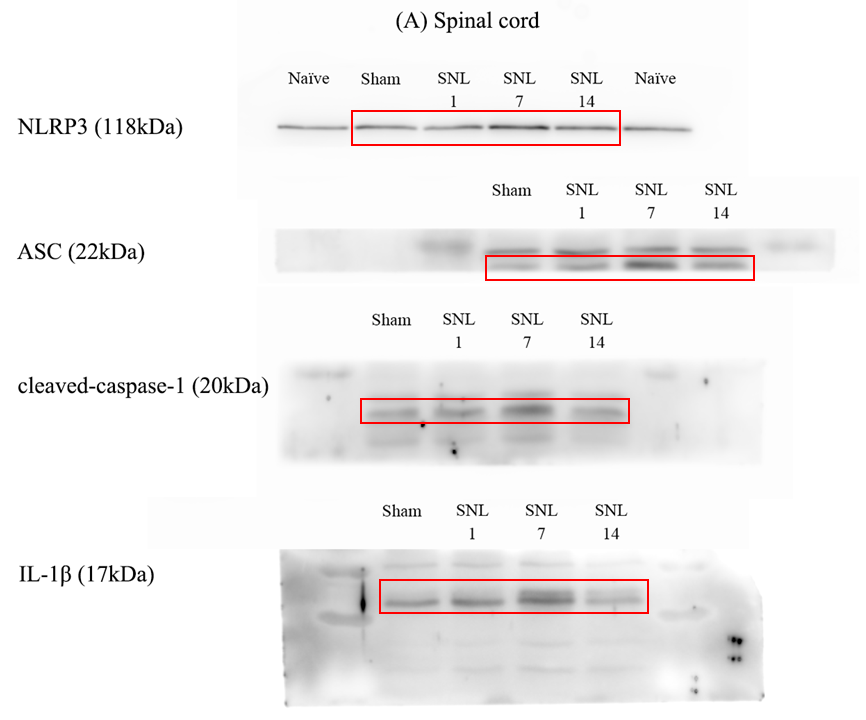
**

**
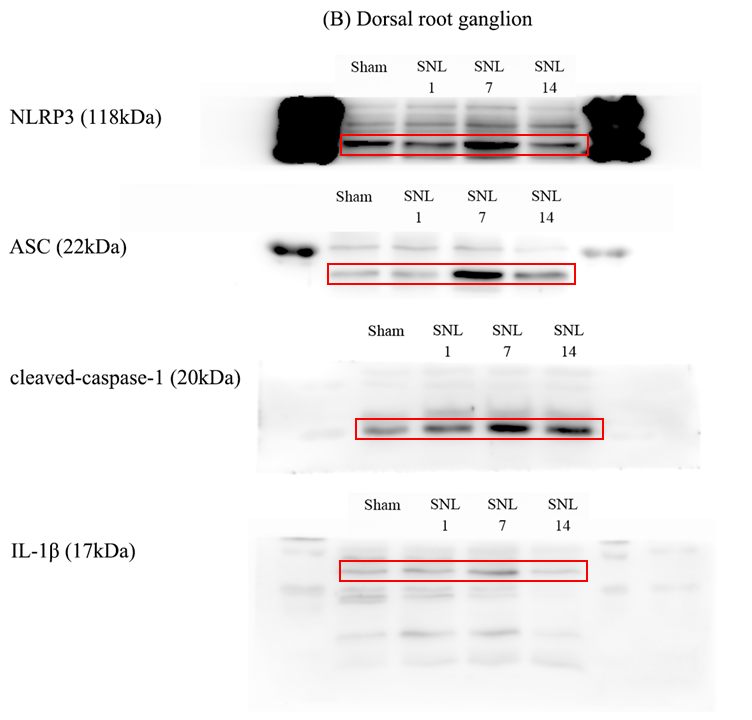
**

**Supplementary figure 4-edges:** Original Western blotting images of spinal cord (A) and dorsal root ganglion (B) of Fig. 4 of the Result section. The rectangular boxes outlined in red highlight the cropped portions for clarity and focus.
