# Supplementary material for: Intrathecal gastrodin alleviates allodynia in a rat spinal nerve ligation model through NLRP3 inflammasome inhibition
Source: BMC Complement Med Ther. 2024 Jun 4;24:213. doi: 10.1186/s12906-024-04519-w (PMC11149323; doi:10.1186/s12906-024-04519-w)
Supplement: Supplementary file 3 — Supplementary Material 3 [file 12906_2024_4519_MOESM3_ESM.docx]

**
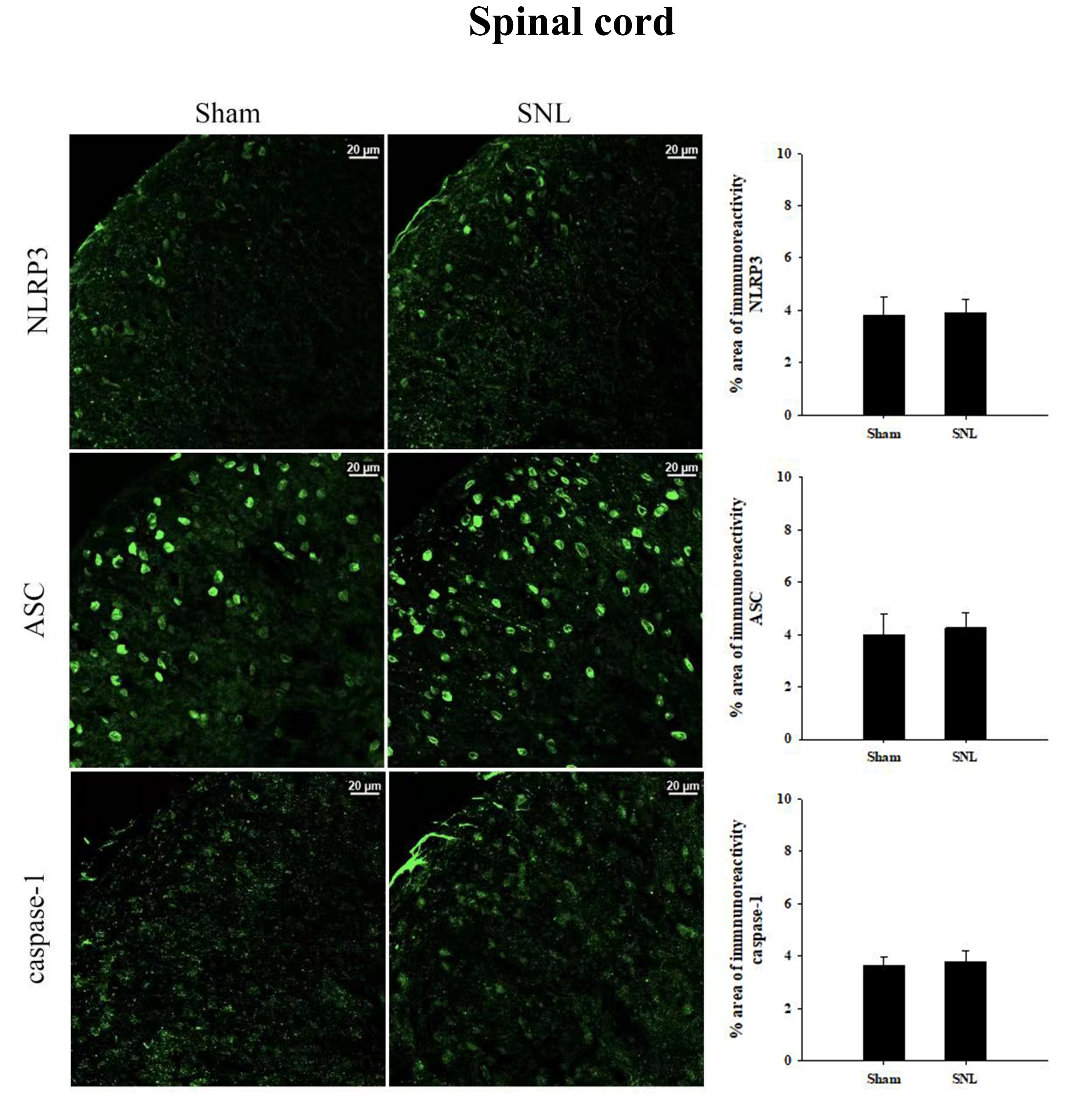
**

**Supplementary figure 5-contralateral:** Images captured from the contralateral side of the spinal cord dorsal horn corresponding to Figure 5. No significant increase in NLRP3 inflammasome expression is observed in the contralateral side in animals of L5/6 spinal nerve ligation (SNL).
