# Supplementary material for: Intrathecal gastrodin alleviates allodynia in a rat spinal nerve ligation model through NLRP3 inflammasome inhibition
Source: BMC Complement Med Ther. 2024 Jun 4;24:213. doi: 10.1186/s12906-024-04519-w (PMC11149323; doi:10.1186/s12906-024-04519-w)
Supplement: Supplementary file 4 — Supplementary Material 4 [file 12906_2024_4519_MOESM4_ESM.docx]

**
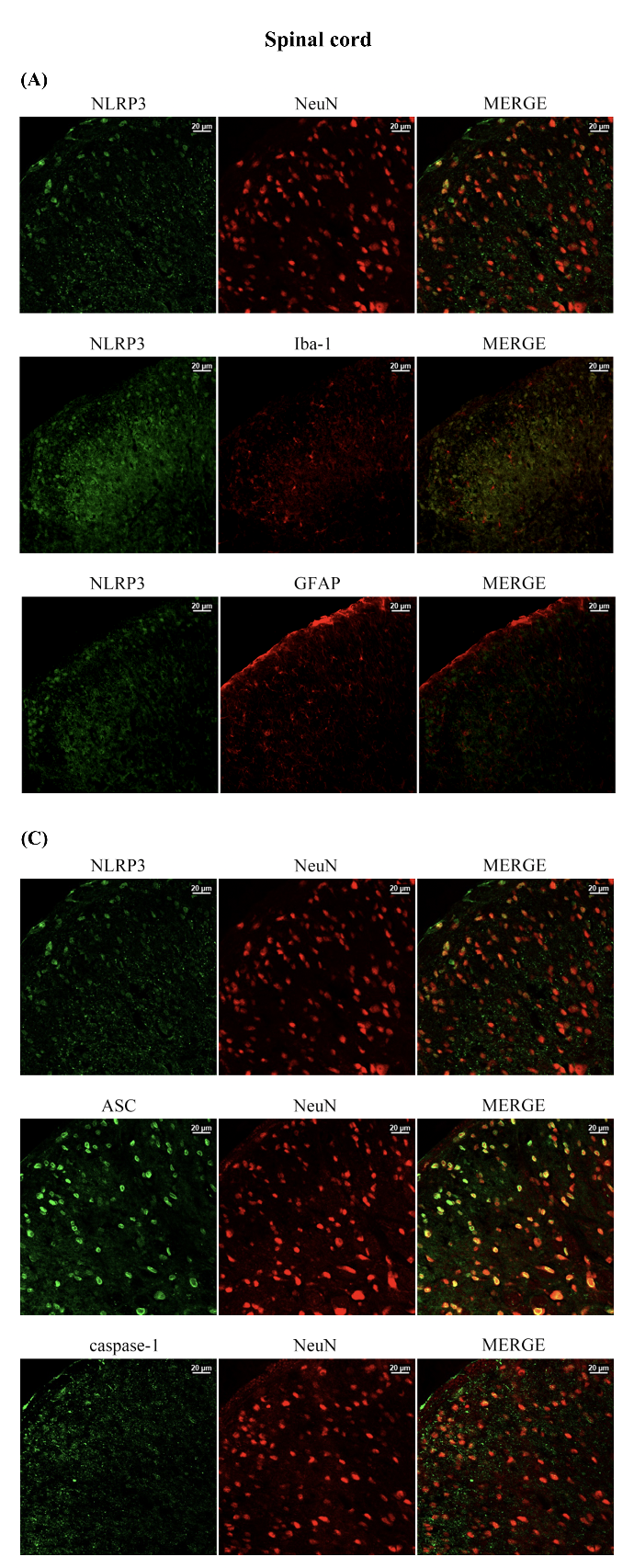
**

**Supplementary figure 6A-contralateral & 6C-contralateral:** Cellular expression of the NLRP3 inflammasome in the spinal dorsal horn contralateral to L5/6 spinal nerve ligation (SNL). The images demonstrate the colocalization of NLRP3 (A, C), ASC (C), and caspase-1 (C) predominantly with NeuN, mirroring the expression pattern depicted in Figure 6.
