# Supplementary material for: Intrathecal gastrodin alleviates allodynia in a rat spinal nerve ligation model through NLRP3 inflammasome inhibition
Source: BMC Complement Med Ther. 2024 Jun 4;24:213. doi: 10.1186/s12906-024-04519-w (PMC11149323; doi:10.1186/s12906-024-04519-w)
Supplement: Supplementary file 5 — Supplementary Material 5 [file 12906_2024_4519_MOESM5_ESM.docx]

**
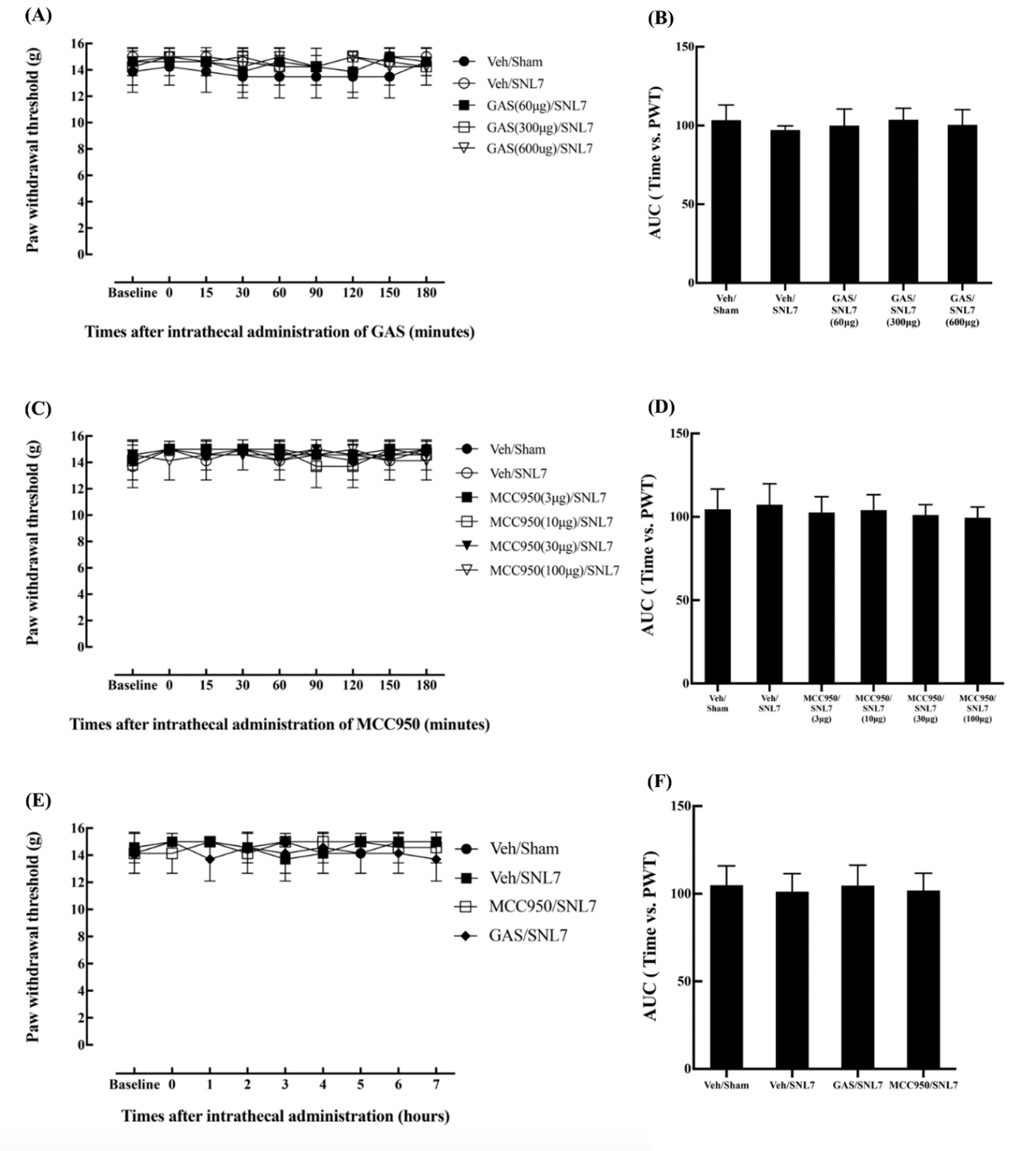
**

**Supplementary figure 7-von Frey-contralateral:** No significant changes in paw withdrawal threshold (PWT) and area under the curve (AUC) following intrathecal (IT) treatment of GAS (gastrodin) or MCC950 in the contralateral side of L5/6 spinal nerve ligation (SNL). The images illustrate the time course of PWT (A, C, E) and corresponding AUC (B, D, F) in contralateral paws of the same animals as depicted in Figure 7.
